# Preparation of antibacterial and osteoconductive 3D-printed PLGA/Cu(I)@ZIF-8 nanocomposite scaffolds for infected bone repair

**DOI:** 10.1186/s12951-020-00594-6

**Published:** 2020-02-27

**Authors:** Fei Zou, Jianyuan Jiang, Feizhou Lv, Xinlei Xia, Xiaosheng Ma

**Affiliations:** Department of Orthopaedics, Huashan Hospital, Fudan University, No. 12 Wulumuqi Zhong Road, Shanghai, 200040 China

**Keywords:** ZIF-8, Three-dimensional printing, Biomaterial scaffold, Antibacterial property, Osteoblastic differentiation

## Abstract

**Background:**

The repair of large bone defects is a great challenge in clinical practice. In this study, copper-loaded-ZIF-8 nanoparticles and poly (lactide-*co*-glycolide) (PLGA) were combined to fabricate porous PLGA/Cu(I)@ZIF-8 scaffolds using three-dimensional printing technology for infected bone repair.

**Methods:**

The surface morphology of PLGA/Cu(I)@ZIF-8 scaffolds was investigated by transmission electron microscopy and scanning electron microscopy. The PLGA/Cu(I)@ZIF-8 scaffolds were co-cultured with bacteria to determine their antibacterial properties, and with murine mesenchymal stem cells (MSCs) to explore their biocompatibility and osteoconductive properties. The bioactivity of the PLGA/Cu(I)@ZIF-8 scaffolds was evaluated by incubating in simulated body fluid.

**Results:**

The results revealed that the PLGA/Cu(I)@ZIF-8 scaffolds had porosities of 80.04 ± 5.6% and exhibited good mechanical properties. When incubated with H_2_O_2_, Cu(I)@ZIF-8 nanoparticles resulted generated reactive oxygen species, which contributed to their antibacterial properties. The mMSCs cultured on the surface of PLGA/Cu(I)@ZIF-8 scaffolds were well-spread and adherent with a high proliferation rate, and staining with alkaline phosphatase and alizarin red was increased compared with the pure PLGA scaffolds. The mineralization assay showed an apatite-rich layer was formed on the surface of PLGA/Cu(I)@ZIF-8 scaffolds, while there was hardly any apatite on the surface of the PLGA scaffolds. Additionally, in vitro, *Staphylococcus aureus* cultured on the PLGA/Cu(I)@ZIF-8 scaffolds were almost all dead, while in vivo inflammatory cell infiltration and bacteria numbers were dramatically reduced in infected rats implanted with PLGA/Cu@ZIF-8 scaffolds.

**Conclusion:**

All these findings demonstrate that PLGA/Cu(I)@ZIF-8 scaffolds possess excellent antibacterial and osteoconductive properties, as well as good biocompatibility and high bioactivity. This study suggests that the PLGA/Cu(I)@ZIF-8 scaffolds could be used as a promising biomaterial for bone tissue engineering, especially for infected bone repair.

## Background

The repair of large bone defects caused by various factors such as trauma, orthopaedic surgeries, tumor resection and infection is still a great challenge in clinical practice. In such cases, the self-healing ability of the bone is not sufficient for bone defect repair without effective external intervention, which is known as bone tissue engineering. However, there are no satisfactory effective biomaterials for use in bone tissue engineering, especially for the repair of infected bone [1]. Thus, optimization of biomaterials is necessary to address this unmet need.

Poly (lactide-*co*-glycolide) (PLGA), polycaprolactone (PCL) and polylactic acid (PLA) are the most used polymer biomaterials for fabricating bone tissue engineering scaffolds [2, 3]. Among these polymer biomaterials, PLGA has characteristics of biodegradability and biocompatibility [3]. PLGA does not have any side effects when applied in medical or biological materials. Its degradation products, lactic acid and glycolic acid, are metabolic products and can be cleared by natural metabolic pathways. To date, around 20 injectable PLGA-based formulations have been approved by the Food and Drug Administration of the USA [4]. Therefore, in the present study, PLGA was selected as the primary raw biomaterial for fabrication of composite scaffolds.

Copper has been demonstrated to have effective antibacterial properties against various bacteria, including *Staphylococcus aureus* [5] and *Escherichia coli* [6], the most common causative microorganisms in bone infections. 317L stainless steel containing copper possessed strong antibacterial activity and biocompatibility, and might be a potential biomaterial for use in implant-related infections [7]. Based on the antibacterial and bone-forming activity of copper, copper coated Ti6Ai4V nails have also been studied to treat implant-related infections [8]. Zinc ions are also known to contribute to antibacterial and osteoconductive effects [9–14]. For example, zinc (Zn)-doped mesoporous hydroxyapatite microspheres/collagen (Zn-MHMs/Coll) scaffolds enhanced osteogenic differentiation of rat bone marrow-derived mesenchymal stem cells and accelerated the formation and healing of bone compared to pure Coll scaffolds or MHMs/Coll scaffolds [9]. The addition of Cu–Zn nanoparticles into chitosan/nano-hydroxyapatite scaffolds conferred antibacterial and osteoproliferative properties on the scaffolds [10], while Zn-containing borosilicate bioactive glass (BG-Zn) scaffolds promoted osteogenesis of human bone marrow-derived stem cells [11]. Compared to pure BG scaffolds, BG-Zn scaffolds encouraged bone tissue regeneration when implanted in rat calvarial defects [11]. Recently, zinc-based zeolitic-imidazolate-frameworks (ZIF-8) were reported to have an excellent antibacterial effect in an infected wound model. Xu et al. [15] reported that ZIF-8-derived carbon nanospheres containing zinc-centered porphyrin-like structures (PMSC) exhibited excellent antibacterial effects and promoted wound healing in an infected wound model. This antibacterial property is attributed to the presence of the zinc sites of ZIF-8. Thus, a combination of copper ions and ZIF-8 nanoparticles were selected to modify the properties of PLGA biomaterials.

Scaffolds are important in bone tissue engineering [16]. In recent decades, three-dimensional (3D) printing technology as emerged and attracted attention due to its user reproducibility and friendliness. It is feasible to fabricate 3D interconnected porous scaffolds precisely and rapidly using computer-aided design and manufacturing [16, 17]. So far, numerous 3D-printed scaffolds have been reported and applied in medicine, such as 3D-printed silk fibroin/hydroxypropyl methyl cellulose scaffolds for facilitating tracheal epithelial regeneration [18], 3D-printed quaternized chitosan-grafted polylactide-*co*-glycolide/hydroxyapatite scaffolds for repairing bone defects [19] and 3D-printed polycaprolactone scaffolds for total ear reconstruction [20].

In the present study, PLGA/Cu(I)@ZIF-8 scaffolds were prepared by 3D printing technology. The antibacterial and osteoconductive properties of the novel PLGA/Cu(I)@ZIF-8 scaffolds were investigated in vitro. We hypothesized that combining PLGA and Cu(I)@ZIF-8 nanoparticles in a 3D-printed scaffold could provide great advantages in terms of antibacterial and osteoconductive efficacy.

## Materials and methods

### Preparation of PLGA/Cu(I)@ZIF-8

First, 2-methylimidazole (1314 mg) and Zn(NO_3_)_2_⋅6H_2_O (594 mg) were each dissolved in 30 mL methanol under ultrasound for 10 min to form clear solutions, respectively. Then, the solutions were added together under stirring, and the final mixture was stirred constantly at 35 °C for 4 h to form ZIF-8. The obtained product was collected by centrifugation, followed by washing with methanol three times and drying at 60 °C under vacuum overnight.

Cu(I)@ZIF-8 was prepared by a simple adsorption technique. Typically, ZIF-8 (160 mg) and cupric chloride (50 mg) were mixed in 5 mL of DDH_2_O. The mixture was stirred at room temperature for 6 h and then added dropwise into 20 mL of glutathione solution (10 mg/mL) at pH 7.4. Excess cupric ions and glutathione were removed by centrifugation at 12,000 rpm for 15 min. The resulting Cu(I)@ZIF-8 nanoparticles were freeze-dried for future use.

PLGA particles were purchased from Jinan Daigang Biological Technology Co. LTD (molecular weight = 200,000 Da; LA/GA = 75/25). The PLGA/Cu(I)@ZIF-8 scaffolds (diameter = 1 cm, height = 0.9 mm) were fabricated layer-by-layer according to the method described in a previous report [19, 21] using a 4th generation 3D Biplotter (EnvisionTEC GmbH, Germany). Briefly, the PLGA solution was prepared by dissolving PLGA particles in 1,4-dioxane. The above Cu(I)@ZIF-8 nanoparticles were added to the PLGA solution and mixed sufficiently by stirring vigorously for 12 h. The mass ratio of Cu(I)@ZIF-8 and PLGA was 1:9. The mixture of PLGA and Cu(I)@ZIF-8 nanoparticles was heated up to 150 °C and extruded from computer-driven dispensers under a pressure of 110 kPa at a speed of 18 mm/s. Finally, the 3D-printed PLGA/Cu(I)@ZIF-8 scaffolds were obtained by drying for 2 days at 37 °C. Pure PLGA scaffolds without Cu(I)@ZIF-8 nanoparticles were fabricated as controls.

### Characterization of Cu(I)@ZIF-8 nanoparticles and PLGA/ Cu(I)@ZIF-8 scaffolds

The surface morphology of Cu(I)@ZIF-8 nanoparticles was observed by transmission electron microscopy (TEM; JEM-2100, Jeol Ltd., Tokyo, Japan). The Multi-Angle static Light Scattering (DAWN HELEOS II; Wyatt Technology Corp., Santa Barbara, CA, USA) was used to measure the particle size of the Cu(I)@ZIF-8 nanoparticles. The surface morphology of scaffolds was observed by digital camera and scanning electron microscopy (SEM; JSM-7001F, eol Ltd.). The pore size of the scaffolds was measured using Image J. The porosity of the scaffolds was detected based on Archimedes’ Principle with a specific gravity bottle (Hubbard) according to the previously reported method [22] (24441182). The mechanical strength of the scaffolds was tested using a mechanical test machine (Instron 5567; Instron, Norwood, MA, USA) with a maximum force setting of 2000 N and a rate of 10 mm/min.

To assess the generation of reactive oxygen species by Cu(I)ZIF-8, different concentrations of Cu(I)ZIF-8 (5 and 10 μg/mL) were mixed with H_2_O_2_ and 3,3′,5,5′-tetramethylbenzidine. The color change represented the generation of reactive oxygen species. The spectral parameters of Cu(I)ZIF-8 nanoparticles were determined by electron spin resonance (ESR) spectroscopy on a Bruker EMX EPR spectrometer (Bruker MicroCT, Kontich, Belgium) and ultraviolet visible (UV–Vis) spectroscopy on a Lambda Bio 40 spectrometer (Perkin Elmer, Waltham, MA, USA).

Additionally, the concentration of Cu(I) ions released from PLGA/Cu(I)@ZIF-8 scaffolds was analyzed in simulated body fluid (SBF) solution at 37 °C by inductively coupled plasma mass spectrometry.

### Isolation and culture of murine mesenchymal stem cells (MSCs)

Murine mesenchymal stem cells (MSCs) were isolated from mouse tissues. The MSCs were cultured in Dulbecco’s modified Eagle’s medium (Invitrogen, Carlsbad, CA) supplemented with 10% fetal bovine serum and 1% penicillin/streptomycin mixture and maintained in an atmosphere of 5% CO_2_ and 37 °C.

### CCK-8 assay

The cell proliferation of cultured MSCs was investigated using the CCK-8 method. After culture on the surface of PLGA or PLGA/Cu(I)@ZIF-8 scaffolds for 1, 4 or 7 days, the MSCs were transferred to a 96-well plate. Aliquots of 10 μL CCK-8 reagent were added into the wells and the cells were incubated for 1 h at 37 °C. Then the absorbance (OD) value at 450 nm wavelength was measured. MSCs cultured without scaffolds were used as controls.

### Cell adhesion and spreading

Cell adhesion and spreading were investigated by immunofluorescence analysis. After culture on the surface of PLGA or PLGA/Cu(I)@ZIF-8 scaffolds for 4 h, the MSCs were fixed with 3% paraformaldehyde for 15 min at room temperature. The fixed MSCs were washed with 10 mM PBS/20 mM glycine solution, then permeabilized with 0.05% saponin for 10 min and blocked using 1% bovine serum albumin for 20 min. Subsequently, the MSCs were incubated with TRITC-phalloidin (1:2000; Sigma, St Louis, MO, USA) and DAPI (1:500; Beyotime Institute of biotechnology, Jiangsu, China) at 37 °C for 1 h to stain the actin filaments of the cytoskeleton and cell nuclei, respectively. After washing with PBS, the samples were observed under a confocal laser scanning microscope.

### Cell culture and induction of osteogenesis

The MSCs were divided into three groups (cultured without scaffolds, cultured on the surface of PLGA scaffolds or on PLGA/Cu(I)@ZIF-8 scaffolds). After culture for 24 h, the cells were changed into osteogenic inductive medium containing 10% fetal bovine serum, 1% mixture of streptomycin (100 mg/mL) and penicillin (100 U/mL) 100 nM dexamethasone and 50 μg/mL ascorbic acid. The medium was changed every 2 days.

### Alkaline phosphatase (ALP) staining and ALP activity assay

The mMSCs in the different groups were collected after 14 days of culture in the osteogenic inductive medium and fixed with 4% paraformaldehyde at 4 °C for 10 min. Then, the fixed MSCs were mixed with staining solution (0.1% naphthol-AS-MX phosphate and 0.1 fast red violet LB salt) and incubated at room temperature for 10 min, then washed in PBS and imaged by microscopy.

### Alizarin red (AR) staining

The MSCs in different groups were collected after 14 days of culture in osteogenic inductive medium and fixed with 4% paraformaldehyde at 4 °C for 10 min. Then, 0.5% Alizarin red S/PBS was added to the fixed MSCs and incubated at room temperature for 10 min. After staining, the cells were washed in PBS and imaged by microscopy.

### Mineralization in simulated body fluid (SBF)

The PLGA scaffolds and PLGA/Cu(I)@ZIF-8 scaffolds were incubated in SBF [23] and maintained at 37 °C. The SBF was replaced once a day. After 14 days’ incubation, the PLGA scaffolds and PLGA/Cu(I)@ZIF-8 scaffolds were removed from the SBF and soaked in deionized water overnight to remove soluble inorganic ions.

### Antibacterial assessment

*Staphylococcus aureus* (ATCC 25923) was purchased from the American Type Culture Collection. First, the scaffolds were incubated in 500 μL *S. aureus* suspension (1 × 10^6^ colony forming units (CFUs)/mL) at 37 °C. After 12 h or 24 h of incubation, the bacterial on the surface of the scaffolds were observed by SEM. Then, after staining with a Live/Dead BacLight viability kit (L7012, Thermo Fisher Scientific, Waltham, MA, USA), the scaffolds were observed by confocal laser scanning microscopy (CLSM).

Next, the adhesion *S. aureus* on the surface of scaffolds was detected according to a previously reported method [24]. A volume of 500 μL of *S. aureus* suspension (1 × 10^6^ CFUs/mL) in Mueller–Hinton Broth medium was added to wells containing PLGA or PLGA/Cu@ZIF-8 scaffolds, and incubated for 4 h at 37 °C. Then, the loosely-adherent bacteria on the surface of the scaffolds were gently removed by washing with sterile phosphate-buffered saline (PBS). Ultrasonication (50 Hz for 10 min), which is known to be one of the most effective approaches to thoroughly removing adherent bacteria on the biomaterials [25], was performed to collect the adherent bacteria from the surface of the scaffolds. After tenfold dilution, the collected solutions were plated in quintuplicate onto tryptic soy agar (TSA) and incubated for 24 h at 37 °C. The counts of the colonies on the TSA indirectly reflected the number of CFUs on the surface of scaffolds.

### Subcutaneous implantation model in rats

Sprague Dawley rats (10 weeks old) were used in the animal study. The animal experiments were approved by the Animal Ethics Committee of Huashan Hospital, Fudan University and performed according to the corresponding guidelines. The contaminated PLGA and PLGA/Cu(I)@ZIF-8 scaffolds were prepared by immersing in *S. aureus* suspensions (1 × 10^8^ CFUs/mL), with the PLGA scaffolds immersed in PBS used as control. After anaesthesia by intraperitoneal injection of 1% pentobarbitone sodium (100 mg/kg), a deep pocket was created by making a longitudinal subcutaneous incision of approximately 1.5 cm in length in the left dorsum, and the contaminated scaffolds were inserted into the pocket. The skin incision was sutured closed and the rats were returned to their cages without any antibiotic treatment and given free access to food and water.

### Immunohistochemical staining and histological evaluation

Three days post-surgery, the rats were sacrificed and the tissues surrounding the scaffolds were collected. The tissues were fixed with 4% paraformaldehyde overnight and then embedded in paraffin, followed by cutting into sections. The sections were probed with IL-6 and TNF-α antibodies to characterize the inflammatory response. Hematoxylin–eosin (H&E) staining was performed to assess the inflammatory cell infiltration and Giemsa staining was performed to assess bacterial contamination.

### Statistical analysis

Quantitative data in all the experiments are presented as mean ± standard deviation. One-way analysis of variance was employed to evaluate the significance of any differences. Multiple comparisons were performed by Tukey’s test. p < 0.05 was considered to indicate statistical significance.

## Results and discussion

### Characterization of Cu(I)@ZIF-8 nanoparticles and PLGA/Cu(I)@ZIF-8 scaffolds

**Fig. 1:**
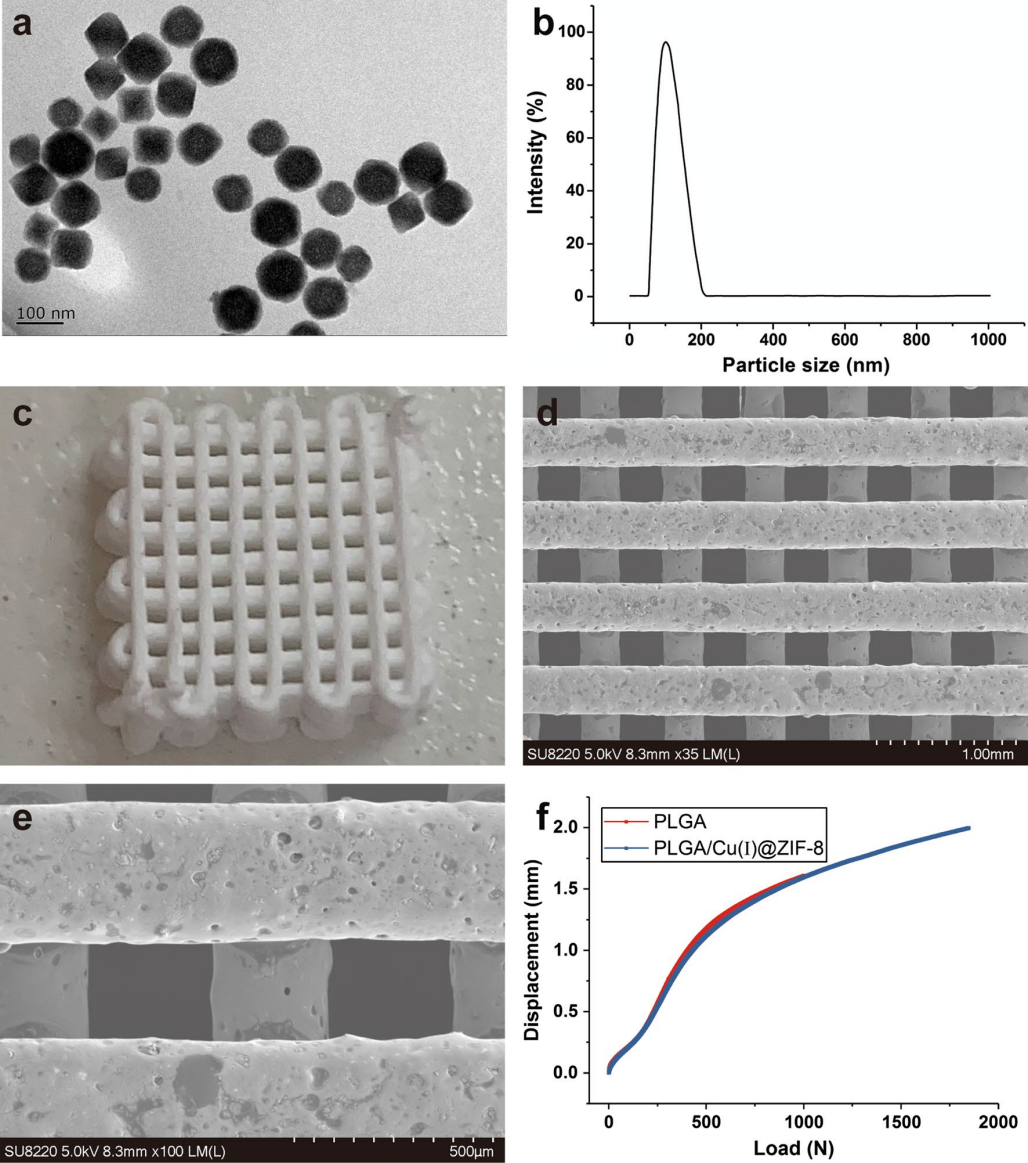
Characterization of Cu(I)@ZIF-8 nanoparticles and PLGA/Cu(I)@ZIF-8 scaffolds. **a** The transmission electron microscope (TEM)image of Cu(I)@ZIF-8 nanoparticles. **b** Particle size distribution of Cu(I)@ZIF-8 nanoparticles. The digital image (**c**) and TEM images (**d**, **e**) of PLGA/Cu(I)@ZIF-8 scaffolds. **f** The load–displacement curve of PLGA scaffolds and PLGA/Cu(I)@ZIF-8 scaffolds

The morphology of Cu(I)@ZIF-8 nanoparticles was observed by TEM. The images revealed that the Cu(I)@ZIF-8 nanoparticles had a similar shape (Fig. 1a) with an average diameter of approximately 85 ± 9.67 nm (Fig. 1b).

The surface morphology of PLGA/Cu(I)@ZIF-8 scaffolds was observed using a digital camera (Fig. 1c) and SEM (Fig. 1d, e). The PLGA/Cu(I)@ZIF-8 scaffolds had a macroporous morphology with a pore size of approximately 350 ± 25 μm and porosity of 80.04 ± 5.6%; while the pure PLGA scaffolds with a pore size of 310 ± 32 μm and porosity of 76 ± 9.2%. The mechanical strength of the PLGA scaffolds and PLGA/Cu(I)@ZIF-8 scaffolds were compared. As shown in Fig. 1f, both PLGA scaffolds and PLGA/Cu(I)@ZIF-8 scaffolds had good mechanical properties with compressive strength of 14.6 ± 4.9 MPa and 17.8 ± 3.6 MPa, respectively. The addition of Cu(I)@ZIF-8 nanoparticles did not affect the mechanical properties of the scaffolds. The scaffolds were integrated and did not fracture even at high load of almost 2000 N, demonstrating the good mechanical strength of PLGA/Cu(I)@ZIF-8 scaffolds.

**Fig. 2:**
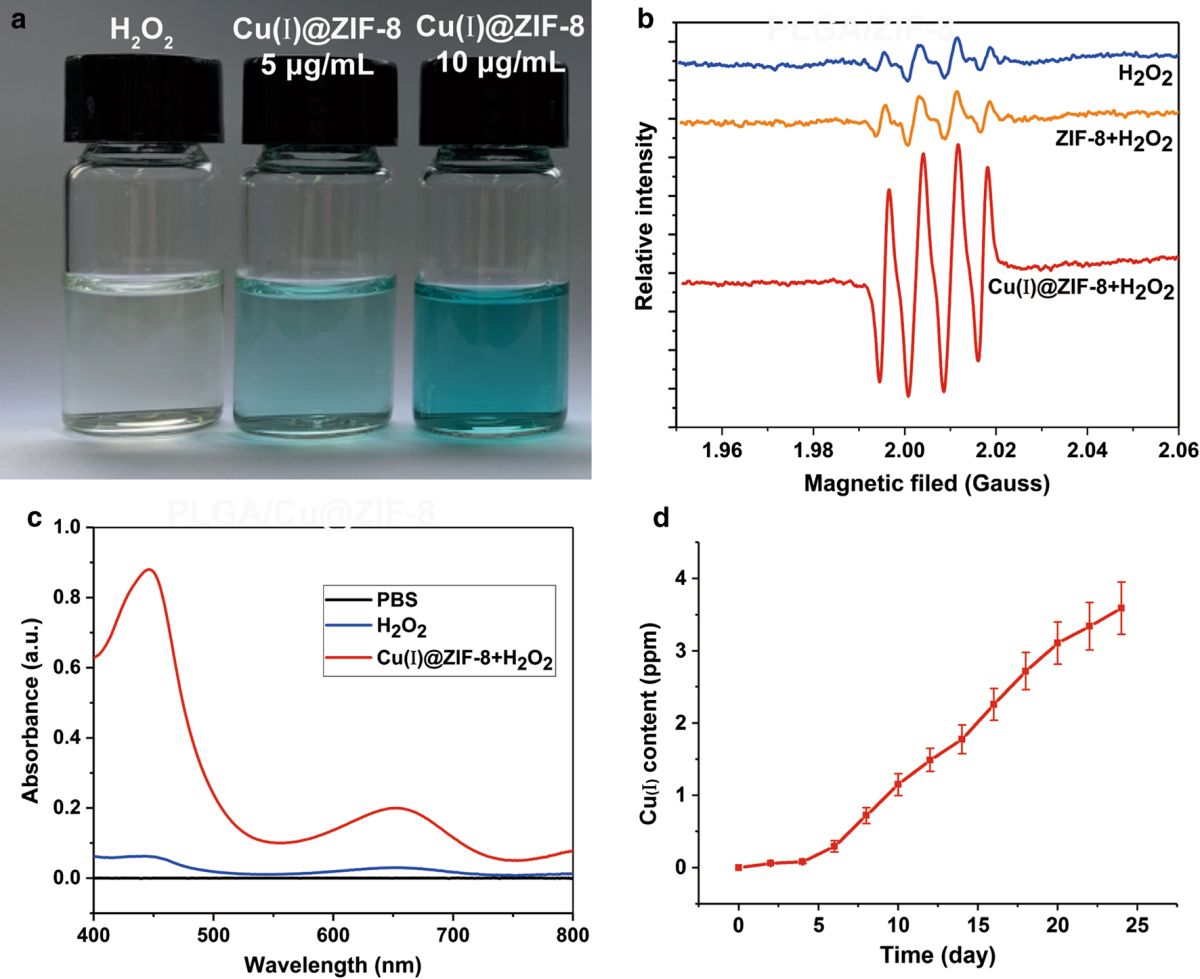
Spectrum analysis of Cu(I)ZIF-8 nanoparticles and Cu(I) ions release from PLGA/Cu(I)ZIF-8 scaffolds. **a** The color change of different concentration of Cu(I)ZIF-8 nanoparticles. **b** Electron spin resonance spectrum of Cu(I)ZIF-8 nanoparticles. **c** Ultraviolet visible spectroscopy spectrum of Cu(I)ZIF-8 nanoparticles. **d** The release curve of Cu(I) ions from PLGA/Cu(I)ZIF-8 scaffolds over time by inductively coupled plasma mass spectrometry (ICP-MS) analysis

Figure 2a shows the color change of Cu(I)@ZIF-8 nanoparticles incubated with H_2_O_2_ and 3,3′,5,5′-tetramethylbenzidine. More reactive oxygen species were generated by 10 μg/mL Cu(I)@ZIF-8 nanoparticles compared with 5 μg/mL Cu(I)@ZIF-8 nanoparticles. ESR and UV–Vis spectra revealed that, compared with the pure ZIF-8 nanoparticles, the Cu(I)@ZIF-8 nanoparticles generated more reactive oxygen species, which contributed to their antibacterial properties (Fig. 2b, c) [26, 27].

The concentration of Cu(I) ions, the antibacterial components, released from PLGA/Cu(I)@ZIF-8 scaffolds was analyzed by ICP-MS. As shown in Fig. 2d, significantly more Cu(I) ions were released from PLGA/Cu(I)@ZIF-8 scaffolds on day 6. The release curve revealed that the Cu(I) ions were slowly and continuously released from PLGA/Cu(I)@ZIF-8 scaffolds over time. The concentration of copper ions in the SBF solution was 3.59 ± 0.36 ppm after incubation for 24 days.

### Biocompatibility of PLGA/Cu(I)@ZIF-8 scaffolds

**Fig. 3:**
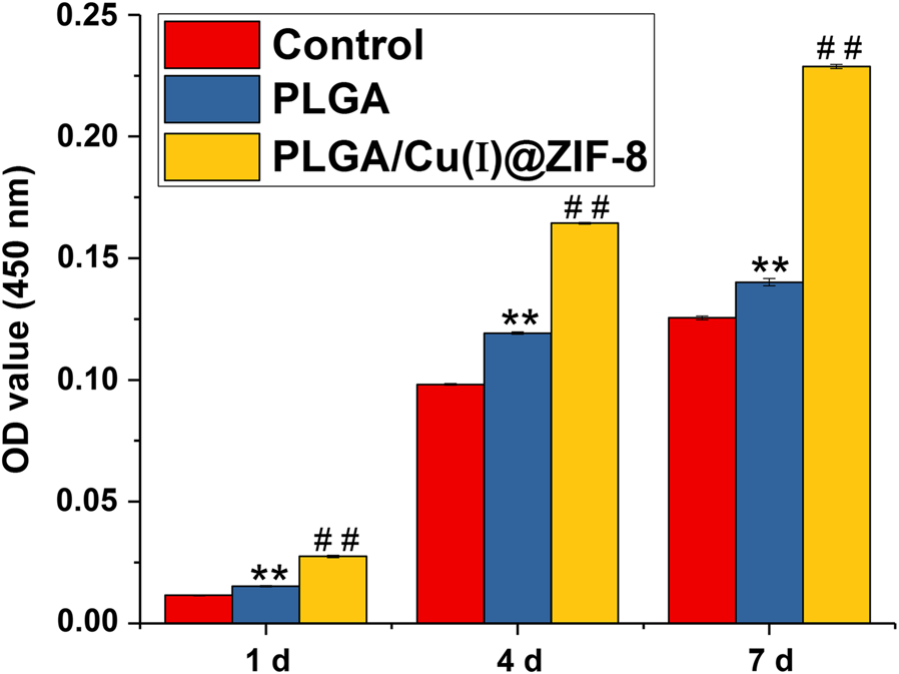
The cell proliferation of murine mesenchymal stem cells (MSCs) on the surface of PLGA scaffolds and PLGA/Cu(I)@ZIF-8 scaffolds after 1 day, 4 days and 7 days. **p < 0.001 compared with the control group; ^##^p < 0.001 compared with the control group and the PLGA scaffolds group

MSCs cultured on the surface of PLGA scaffolds had higher cell viability than cells grown without scaffolds (Fig. 3). Moreover, compared with MSCs cultured on the surface of PLGA scaffolds, cells cultured on the surface of PLGA/Cu(I)@ZIF-8 scaffolds had significantly enhanced cell viability. This result indicated that 3D-printed scaffolds facilitated cell proliferation, and the addition of Cu(I)@ZIF-8 nanoparticles promoted cell proliferation further.

**Fig. 4:**
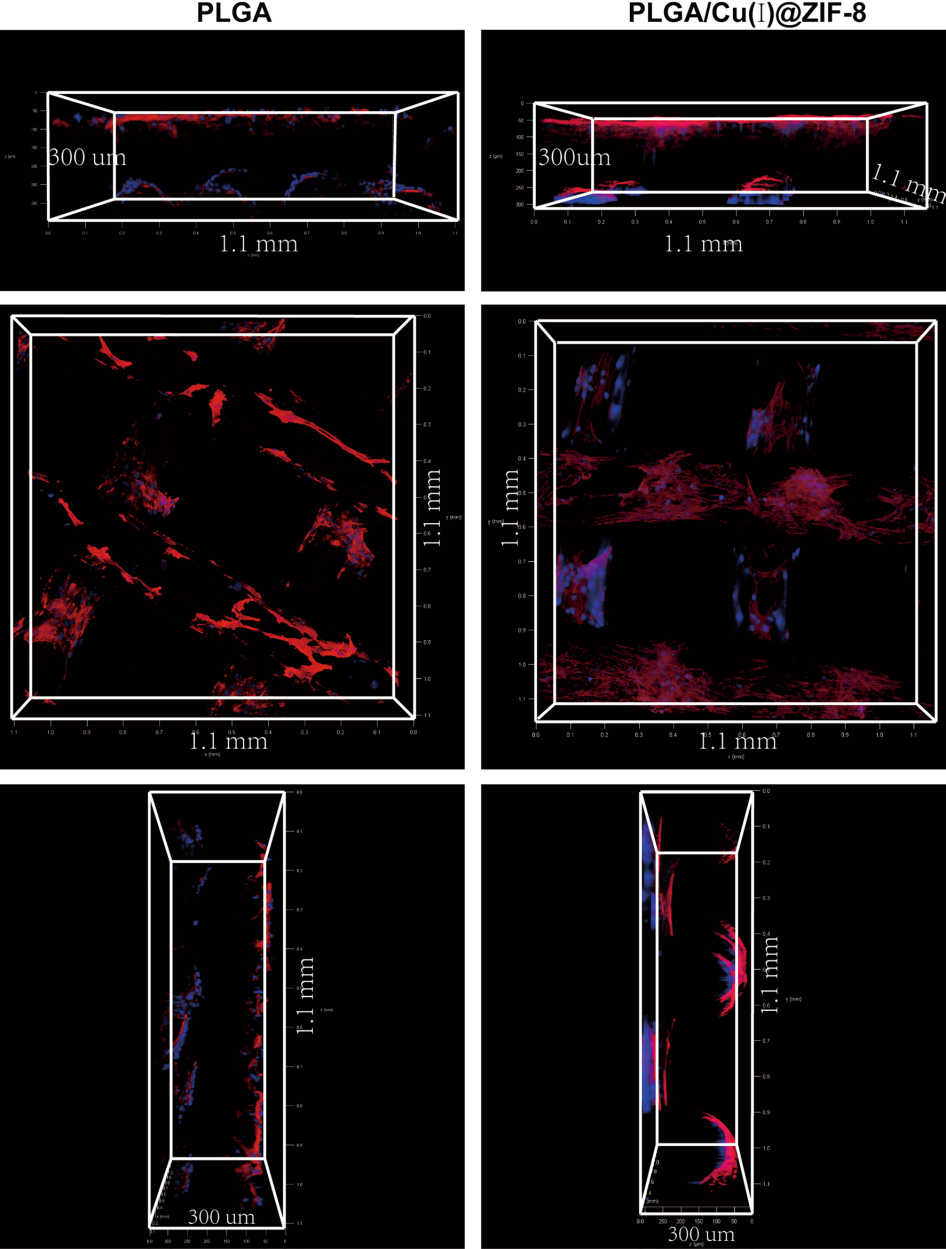
MSCs adhering to the surface of PLGA scaffolds and PLGA/Cu(I)@ZIF-8 scaffolds

Cell adhesion was evaluated by immunofluorescence staining. Figure 4 shows representative images of MSCs adhering to the surface of PLGA and PLGA/Cu(I)@ZIF-8 scaffolds. The blue fluorescence represents cell nuclei stained with DAPI, while the red fluorescence represents actin filaments of the cytoskeleton stained with TRITC-phalloidin. MSCs cultured on the surface of PLGA/Cu(I)@ZIF-8 scaffolds were more spread and adhered better than those on the surface of pure PLGA scaffolds. Moreover, the number of adherent MSCs was obviously greater in the case of PLGA/Cu(I)@ZIF-8 scaffolds.

An ideal scaffold for bone tissue engineering should meet the following criteria: low or no cytotoxicity, good biocompatibility, biodegradation, adequate physical properties and mechanical properties [16, 28]. Physical properties of the biomaterial scaffolds, such as porosity and pore size have been demonstrated to relate to the biocompatibility of cells, cell proliferation and cell attachment, resulting in bone formation and better repair of bone and osteochondral defects [29, 30]. In this study, the PLGA/Cu(I)@ZIF-8 scaffolds had interconnected pores with porosity of 80.04 ± 5.6% and pore size of 350 μm. Moreover, the PLGA/Cu(I)@ZIF-8 scaffolds also had good mechanical strength, thus meeting the critical criteria of scaffolds in bone tissue engineering. MSCs cultured on the surface of PLGA/Cu(I)@ZIF-8 scaffolds exhibited good biocompatibility, including higher cell viability, better cell adhesion and cell spreading, compared with cells on the pure PLGA scaffolds. Thus, PLGA/Cu(I)@ZIF-8 had good potential for bone tissue engineering.

### Osteoconductive properties of PLGA/Cu(I)@ZIF-8 scaffolds

**Fig. 5:**
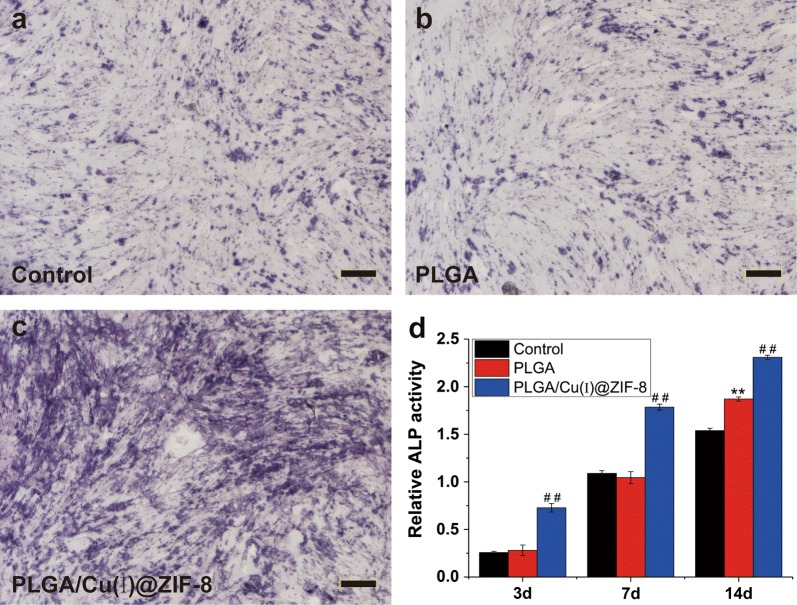
Alkaline phosphatase (ALP) staining (**a**–**c**) and ALP activity (**d**) of MSCs cultured on the surface of PLGA scaffolds and PLGA/Cu(I)@ZIF-8 scaffolds. **p < 0.001 compared with the control group; ^##^p < 0.001 compared with the control group and the PLGA scaffolds group

Alkaline phosphatase (ALP) is an early marker of osteogenic differentiation in the cell mineralization progress [31]. The osteogenic differentiation of MSCs cultured on the surface of PLGA scaffolds or PLGA/Cu(I)@ZIF-8 scaffolds was investigated by ALP staining and ALP activity assay. As shown by ALP staining, a few ALP-positive cells were present among MSCs cultured on PLGA scaffolds, as well as in control MSCs cultured without any scaffolds (Fig. 5a, b). In contrast, ALP staining was extensive in the MSCs cultured on PLGA/Cu(I)@ZIF-8 scaffolds (Fig. 5c). MSCs cultured on the surface of PLGA/Cu(I)@ZIF-8 scaffolds had markedly higher ALP activity compared with the other two groups (Fig. 5d).

**Fig. 6:**
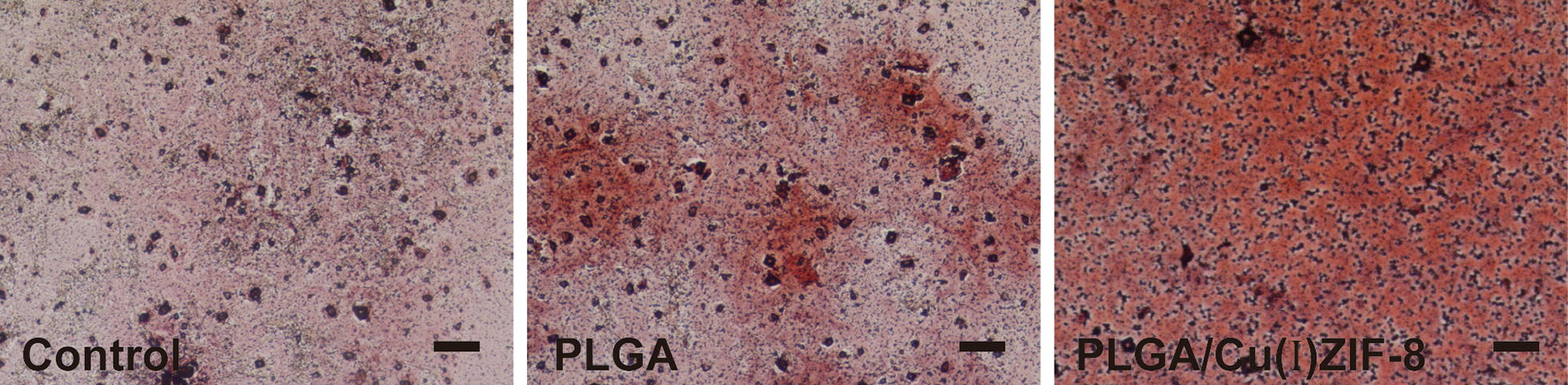
Alizarin red staining (AR) of MSCs cultured on the surface of PLGA scaffolds and PLGA/Cu(I)@ZIF-8 scaffolds

Osteoblastic differentiation is also characterized by calcium deposits. As the main mineral component of the bone matrix, calcium is usually evaluated by alizarin red (AR) staining [31]. As shown in Fig. 6, MSCs cultured on PLGA/Cu(I)@ZIF-8 scaffolds were strongly stained by AR, while MSCs cultured on PLGA scaffolds showed some staining and control cultures without any scaffolds were barely stained. Thus, MSCs co-cultured with PLGA scaffolds in the osteogenic inductive medium had more ALP and AR staining, and higher ALP activity, compared with cells on pure PLGA scaffolds, indicating a positive effect of PLGA/Cu(I)@ZIF-8 scaffolds on osteoblastic differentiation.

### Bioactivity of PLGA/Cu(I)@ZIF-8 scaffolds

**Fig. 7:**
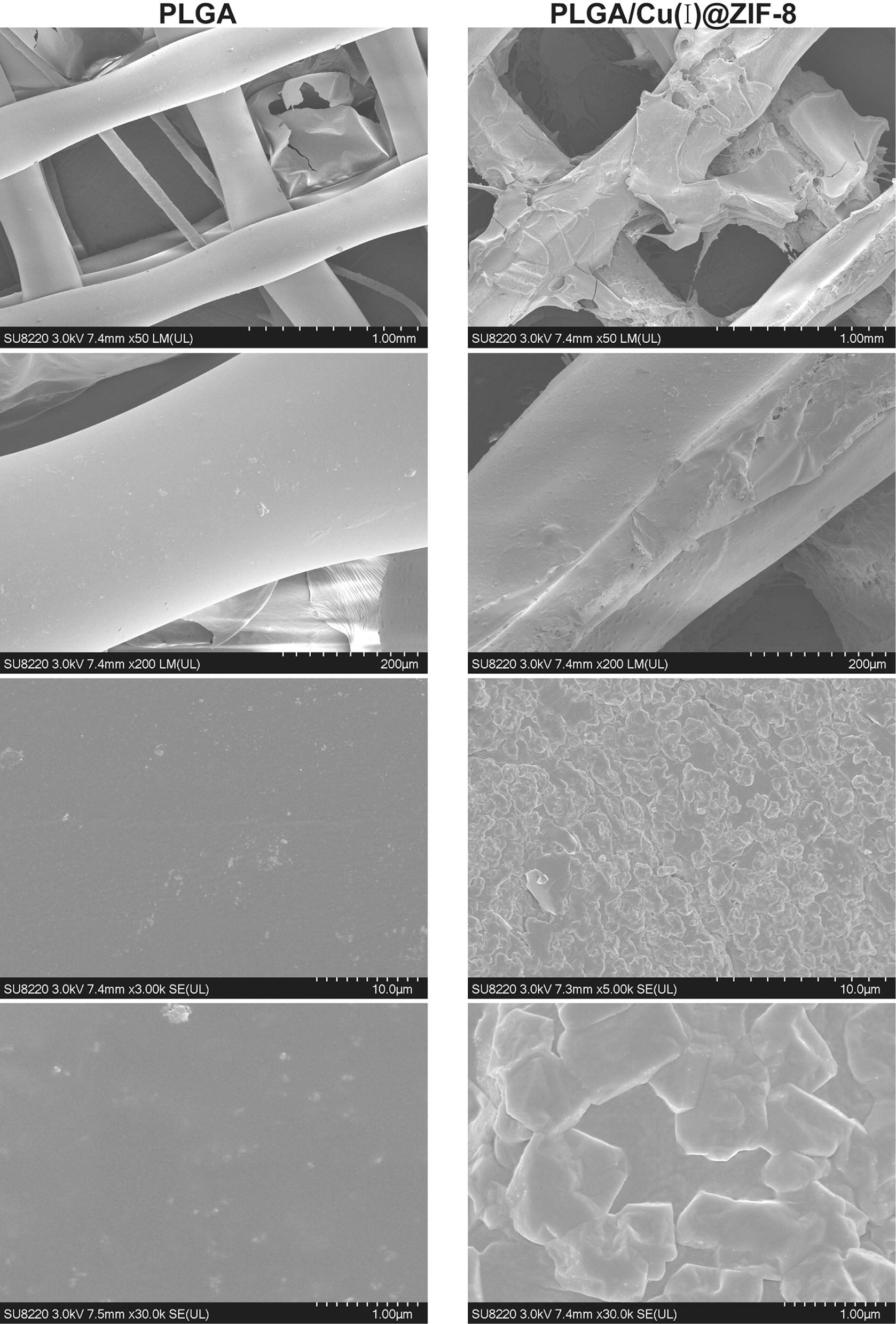
SEM images of PLGA scaffolds and PLGA/Cu(I)@ZIF-8 scaffolds incubated in SBF solution

The bioactivity of PLGA scaffolds and PLGA/Cu(I)@ZIF-8 scaffolds was assessed by mineralization studies in SBF. After incubation in SBF for 14 days, SEM images at different magnifications revealed that an apatite-rich layer formed in PLGA/Cu(I)@ZIF-8 scaffolds, while there was little apatite present in PLGA scaffolds (Fig. 7). The formation of apatite on the surface of materials in SBF has been showed to effectively predict the bone bioactivity of materials [23, 32]. Thus, the apatite-rich layer on the surface of PLGA/Cu(I)@ZIF-8 revealed its high bioactivity.

### Antibacterial assessment of PLGA/Cu(I)@ZIF-8 scaffolds in vitro and in vivo

**Fig. 8:**
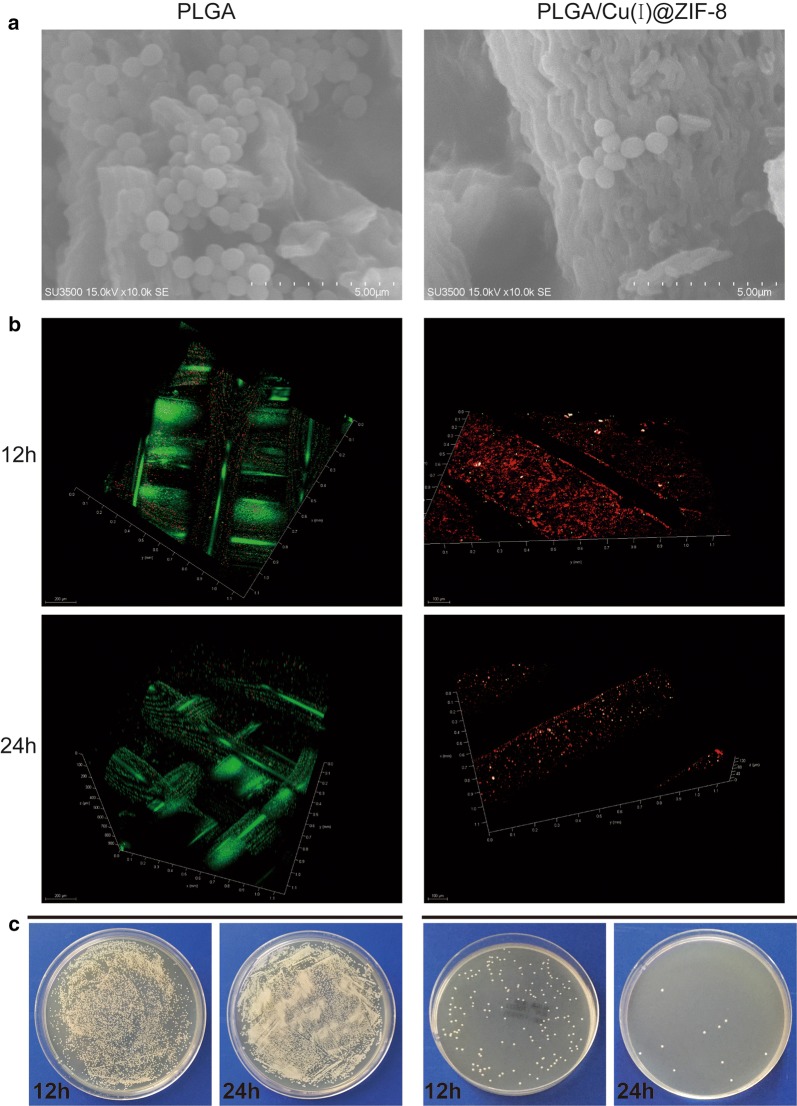
The bacterial growth on the surface of PLGA scaffolds and PLGA/Cu(I)@ZIF-8 scaffolds. **a** Observation of bacteria on the surface of PLGA scaffolds and PLGA/Cu(I)@ZIF-8 scaffolds by SEM. **b** Live and dead bacterial observation on the surface of PLGA scaffolds and PLGA/Cu(I)@ZIF-8 scaffolds after 12 h and 24 h of incubation. **c** Bacterial adhesion on the surface of PLGA scaffolds and PLGA/Cu(I)@ZIF-8 scaffolds was indirectly detected by colony forming assay

**Fig. 9:**
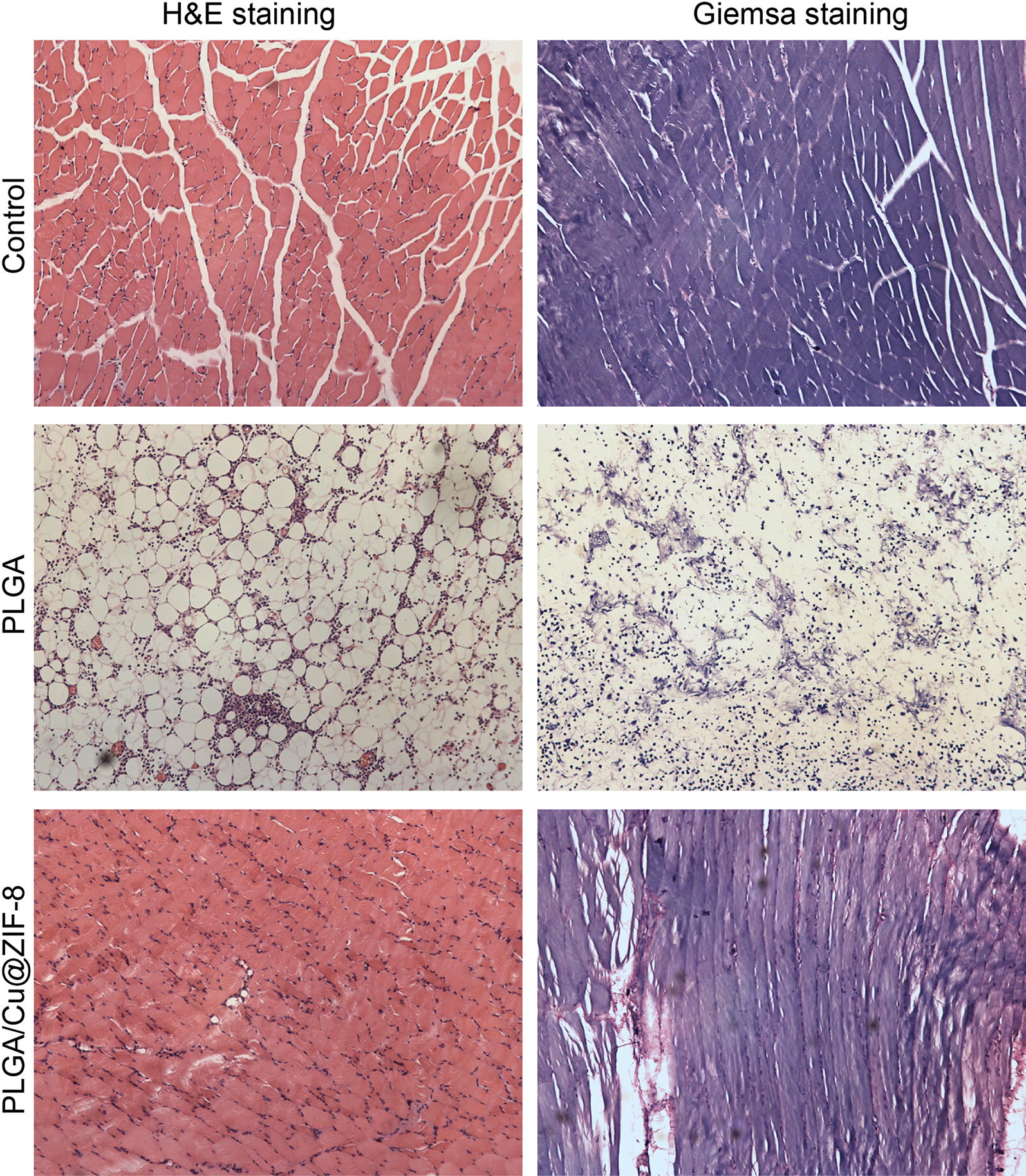
Representative hematoxylin–eosin (H&E) and Giemsa staining images for the soft tissues surrounding the scaffolds at 3 days after implantation

**Fig. 10:**
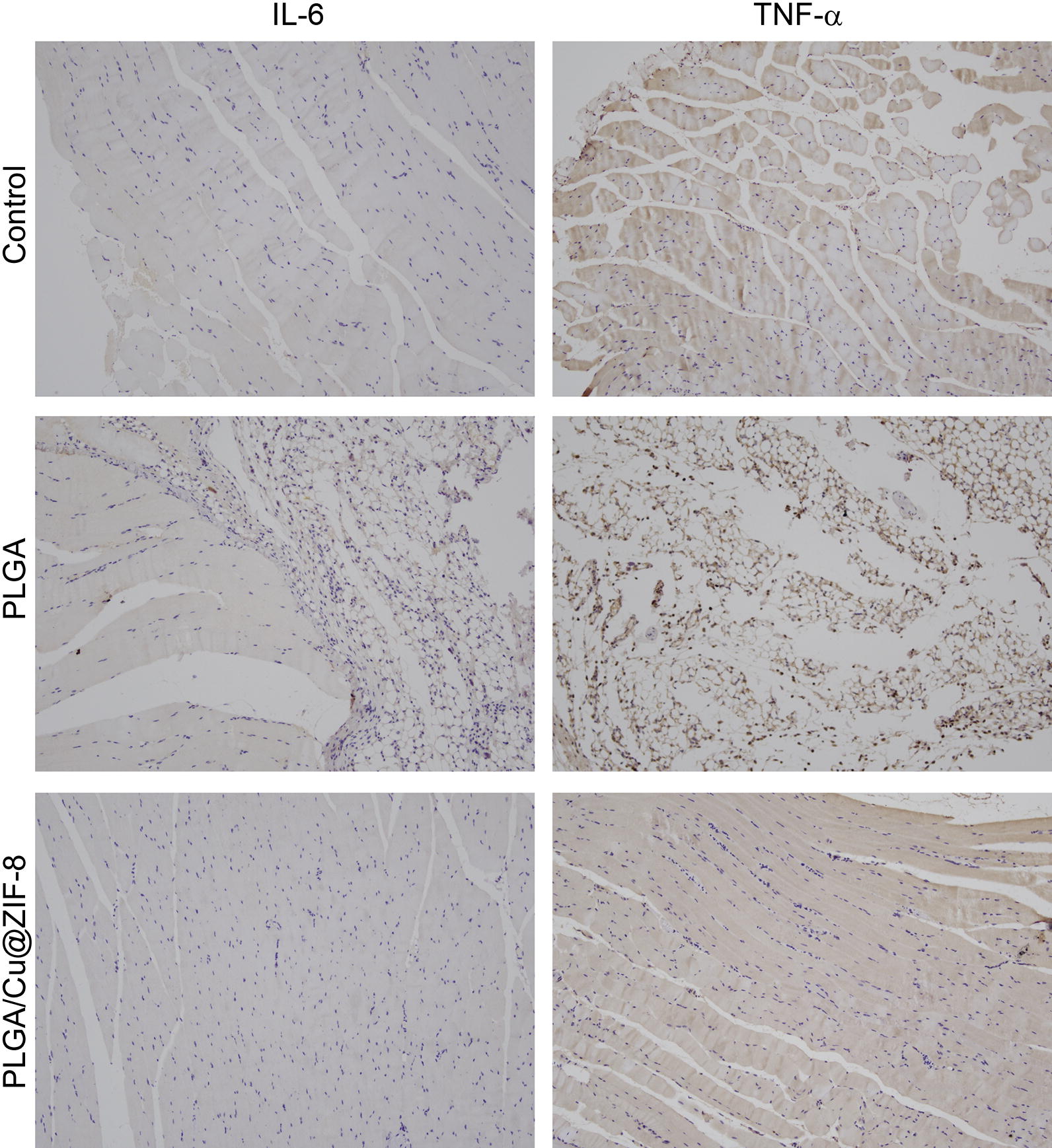
Immunohistochemistry staining of inflammation-related genes IL-6 and TNF-α in the infected tissue with different treatments

*Staphylococcus aureus* is the most common causative microorganism in bone infection [1]. In the present study, we evaluated the in vitro and in vivo antibacterial efficacy of PLGA/Cu(I)@ZIF-8 scaffolds. In vitro, *S. aureus* was seeded onto the surface of PLGA/Cu(I)@ZIF-8 scaffolds. SEM images showed that the number of bacteria on the surface of PLGA/Cu(I)@ZIF-8 scaffolds was obviously reduced compared with those on the surface of PLGA scaffolds (Fig. 8a). Live and dead *S. aureus* were observed by CLSM. After culture for 12 or 24 h, the results of immunofluorescence analysis showed that most of the bacterial cultured on the surface of PLGA scaffolds were alive, while bacteria cultured on the surface of PLGA/Cu(I)@ZIF-8 scaffolds were almost all dead (Fig. 8b). Consistent with the SEM analysis and immunofluorescence study, the number of colonies on TSA was much lower in the PLGA/Cu(I)@ZIF-8 scaffold group compared with the pure PLGA scaffold group (Fig. 8c). Moreover, the number of colonies on TSA in the PLGA/Cu@ZIF-8 scaffold group reduced over time. Thus, the PLGA/Cu(I)@ZIF-8 scaffolds evidently inhibited bacteria growth. All these results indicated that PLGA/Cu(I)@ZIF-8 scaffolds exerted excellent antibacterial effects against *S. aureus*. In vivo, histological evaluation was performed by H&E staining and Giemsa staining. Compared with the control group, in the pure PLGA scaffolds group there was obvious inflammatory cell infiltration, shown by H&E staining, as well as a mass of bacteria as shown by Giemsa-staining (Fig. 9). While the inflammatory cell infiltration and bacteria numbers were both dramatically reduced in the PLGA/Cu@ZIF-8 scaffolds group. Moreover, the infected tissue was reduced in the subcutaneous implantation model treated with PLGA/Cu(I)@ZIF-8 scaffolds, evidenced by a reduction in the IL-6 and TNF-α levels (Fig. 10).

Copper has been demonstrated to have an effective antibacterial effect against various bacteria, including *S. aureus* [5] and *Escherichia coli* [6] and has been used to modify different biomaterials as treatment for implant-related infections [7, 8]. The addition of copper into mesoporous bioactive glass scaffolds or Cu–Zn nanoparticles into chitosan/nano-hydroxyapatite scaffolds conferred antibacterial and osteoproliferative properties on the scaffolds [10, 33], Jin et al. revealed that Zn-implanted titanium had antibacterial effects on *E. coli* and stimulated the spreading activity, adhesion, ALP activity and extracellular matrix mineralization of rat mesenchymal stem cells [14]. Zn-modified TiCaPCON films showed a significantly antibacterial effect against *E. coli* and *S. aureus* strains and good osteoconductive characteristics [13]. In our present study, the PLGA/Cu(I)@ZIF-8 scaffolds provided a continuous release of Cu(I) ions even after 24 days of incubation in SBF solution; while ZIF-8 nanoparticles themselves possessed antibacterial activity [15]. These might be the cause of the effects of PLGA/Cu(I)@ZIF-8 scaffolds on cell proliferation, cell adhesion, cell spreading and osteoblastic activity of MSCs, as well as their effect against *S. aureus*.

## Conclusion

In this study, we developed porous PLGA/Cu(I)@ZIF-8 nanocomposite scaffolds for infected bone repair. The PLGA/Cu(I)@ZIF-8 scaffolds were fabricated by combining Cu(I)@ZIF-8 nanoparticles with PLGA by 3D printing technology. The scaffolds had porosity of 80.04 ± 5.6% and exhibited good mechanical properties. The PLGA/Cu(I)@ZIF-8 scaffolds promoted the proliferation of murine mesenchymal stem cells (MSCs) and facilitated cell adhesion and spreading, as well as significantly inducing osteoblastic differentiation of MSCs. Meanwhile, PLGA/Cu(I)@ZIF-8 scaffolds showed excellent antibacterial characteristic in vitro and in vivo. Overall, our novel 3D-printed PLGA/Cu(I)@ZIF-8 scaffolds demonstrate good potential for bone tissue engineering, especially for the treatment of infected bone defects.

## Data Availability

All data generated or analysed during this study are included in this published article.

## Abbreviations

ZIF-8: Zeolitic-imidazolate-frameworks
PLGA: Poly (lactide-co-glycolide)
MSCs: Mesenchymal stem cells
PCL: Polycaprolactone
PLA: Polylactic acid
Zn-MHMs/Coll: Zinc (Zn)-doped mesoporous hydroxyapatite microspheres/collagen
PMSC: Porphyrin-like structures
3D: Three-dimensional
TEM: Transmission electron microscopy
SEM: Scanning electron microscopy
SBF: Simulated body fluid
OD: Absorbance
PBS: Phosphate-buffered saline
CFUs: Colony forming units
TSA: Tryptic soy agar
H&E: Hematoxylin–eosin
ALP: Alkaline phosphatase
AR: Alizarin red
*S. aureus*: *Staphylococcus aureus*
*E. coli*: *Escherichia coil*
